# Workplace Social and Organizational Environments and Healthy-Weight Behaviors

**DOI:** 10.1371/journal.pone.0125424

**Published:** 2015-04-28

**Authors:** Rachel G. Tabak, J. Aaron Hipp, Christine M. Marx, Ross C. Brownson

**Affiliations:** 1 Prevention Research Center in St. Louis, Brown School, Washington University in St. Louis, St. Louis, Missouri, United States of America; 2 Division of Public Health Sciences, Washington University School of Medicine, St. Louis, Missouri, United States of America; 3 Division of Public Health Sciences and Alvin J. Siteman Cancer Center, Washington University School of Medicine, St. Louis, Missouri, United States of America; Medical University Vienna, AUSTRIA

## Abstract

**Background:**

The workplace is an important setting for health promotion including nutrition and physical activity behaviors to prevent obesity. This paper explores the relationship between workplace social environment and cultural factors and diet and physical activity (PA) behaviors and obesity among employees.

**Methods:**

Between 2012 and 2013, telephone interviews were conducted with participants residing in four Missouri metropolitan areas. Questions included demographic characteristics, workplace socio/organizational factors related to activity and diet, and individual diet and PA behaviors, and obesity. Multivariate logistic regression was used to examine associations between the workplace socio/organizational environment and nutrition, PA, and obesity.

**Results:**

There were differences in reported health behaviors and socio/organizational environment by gender, race, age, income, and worksite size. For example, agreement with the statement the ‘company values my health’ was highest among Whites, older employees, and higher income workers. As worksite size increased, the frequency of reporting seeing co-workers doing several types of healthy behaviors (eat fruits and vegetables, doing PA, and doing PA on breaks at work) increased. In adjusted analyses, employees agreeing the company values my health were more likely to engage in higher PA levels (aOR=1.54, 95% CI: 1.09-2.16) and less likely to be obese (aOR=0.73, 95% CI: 0.54-0.98). Seeing co-workers eating fruits and vegetables was associated with increased reporting of eating at least one vegetable per day (aOR=1.43, 95% CI: 1.06-1.91) and seeing co-workers being active was associated with higher PA levels (aOR 1.56, 95% CI: 1.19-2.05).

**Conclusions:**

This research suggests that social/organizational characteristics of the workplace environment, particularly feeling the company values the workers’ health and to seeing co-workers engaging in healthy behaviors, may be related to nutrition and PA behaviors and obesity. These findings point to the potential for intervention targets including environment and policy changes.

## Introduction

Poor nutrition and inadequate physical activity (PA) are lifestyle behaviors resulting in obesity and a host of chronic diseases [1–4]. The scale of the public health problem necessitates that efforts to promote healthy weight reach large portions of the population; however, existing efforts have had limited success [5, 6], as many have focused on individuals and have targeted behavior change through psychosocial and cognitive–behavioral strategies. Ecologic frameworks highlight the importance of factors beyond the individual level and show how the individuals’ environments can be related to their behaviors [7, 8]. Theories of environmental and health interactions have also suggested that an environment may promote or prevent healthy behaviors through the physical, interpersonal, organizational, and sociocultural characteristics of a setting [8].

Considering the potential for environments to impact health beyond the individual level, worksites may be an effective setting for efforts to promote healthy weight; according to the American Time Use Survey, on average, adults spend 8.8 hours per day in work and work-related activities (http://www.bls.gov/tus/charts/). Researchers have found relationships between workplace supports (e.g., incentives and facilities that support active transportation such as bikelock areas and showers or access to Employee Assistance Programs) and employee health and well-being [9, 10] and health behaviors [11, 12]. In addition to specific workplace supports, the social environment in the workplace can influence employee health. The social environment can influence behavior by “shaping norms, enforcing patterns of social control (which can be health promoting or health damaging); providing or denying opportunities to engage in particular behaviors; and reducing or producing stress,” as well as by “placing constraints on individual choice” [13]. Behavior theories suggest the importance of social norms, including those in the worksite, in determining health behaviors [14–16], and norms have been shown to influence obesity [16] and other health-related behaviors [17]. Surrounding culture and social environment can influence obesity and behavior [15, 18], and the workplace environment is particularly important [19]. Social norms in the workplace, in particular, may influence workplace outcomes such as safety behaviors [20]. However, while studies exploring correlates and interventions in the environmental realm have shown benefits on diet and activity behaviors, research on social environment changes has been more limited [11, 21, 22]. Specifically, the literature showing empirical relationships and precise leverage points for influencing employee health is sparse. One study has investigated the relationship between such social/organizational characteristics as worksite support and co-worker behaviors, however, this was limited to hospital employees in one hospital system [11]. Additionally, previous research has shown that demographic characteristics of workers (e.g., race and gender) and workplace size are associated with diet and activity behaviors [23–29] and/or workplace characteristics [30, 31]. Therefore, these factors need further study. This paper explores the relationship between workplace social environment and cultural factors and diet and PA behaviors and obesity in a large sample of employees from a diverse set of workplace settings across multiple metropolitan areas.

## Materials and Methods

### Design

Study participants were from the Supports at Home and Work for Maintaining Energy Balance (SHOW-ME) study [32], a cross-sectional telephone-survey based study, which aimed to examine associations between residential and worksite environmental and policy influences on energy balance outcomes. The SHOW-ME Study aims to understand the association between obesity and the environments and policies where employed adults live and work. It is part of a national network of research on the relationship between obesity and cancer across the life course [33].

### Sample

This study utilized census tracts in four Missouri metropolitan areas (St. Louis area, Kansas City area, City of Springfield, and City of Columbia) for sampling in order to provide generalizability of Missouri metropolitan areas, variation in the built environment, and representation by racial/ethnic minority and low-income populations. To be included in sampling, a census tract could not have a population density less than 10^th^ percentile of the population density of study areas or more than 50% inhabitants aged 15–24 years. To achieve the desired sample, a multistage stratified sampling procedure allowed for sampling individuals within seven strata. These included: metro size (large vs. small), and within the large metro size are the walkability (low, moderate, and high), and racial/ethnic minority (low vs. high) strata [34]. List-assisted, targeted telephone random-digit-dialing was used to contact and recruit potential participants with landline phone numbers. Prior to the start of the data collection period, the most current data on cell phone use [35, 36] showed that ~27.8% of adults in the US live in households with only wireless phones. This percentage was lower for all of Missouri (22.4%) where all study areas are located, but higher for adults age 34 and younger, for racial and ethnic minorities, and for those living in poverty. Despite these differences, recruitment distribution was monitored throughout the data collection period and did not show any dearth of participants in these categories. There may have been other characteristics of wireless-only households that were not captured through standard demographics, but we attempted to address this potential bias. The first eligible adult from each household to volunteer was included in the sample; only one participant per household was included. The response rate was 15%. Two thousand fifteen participants were recruited in three waves, between April 2012 and April 2013. Inclusion criteria required the participant be: between the ages of 21 and 65 years; employed outside of the home at one primary location; employed for 20 or more hours per week at one site with at least five employees; not pregnant; and no physical limitation to prevent walking or bicycling in the past week.

### Ethics Statement

The study design was approved by the Human Research Protection Office of Washington University in St. Louis. Using an Institutional Review Board-approved telephone script, a trained member of the research team read a description of the research (purpose, general content, risks). The research team member asked for verbal consent from the potential participant and informed the participant of his or her ability to withdraw consent to participate in the research project at any time. Once the participant provided verbal consent, the research team member administered the survey; as such, continued participation in the survey indicates continued consent. This was approved by the Human Research Protection Office.

### Measures

#### Survey Development

The survey tool was developed for this study. The exact wording and options for all items used for the current analysis are available in S1 Appendix. Existing self-reported and environmental assessment instruments were used as the basis for the survey items, as was previous experience of the project team and input from a special Questionnaire Advisory Panel (including researchers from universities in the US and Australia and from the Centers for Disease Control and Prevention) who are experts in survey development, nutrition/food environment, PA, transportation, and worksite environmental intervention, convened especially for this study. Test-retest assessment in a subsample found that reliability coefficients (Spearman correlations for continuous variables, Cohen’s κ statistics for non-ordinal categorical variables, and 1-way random intraclass correlation coefficients for ordinal categorical variables) ranged from 0.41 to 0.97, with 80% of items having reliability coefficients of more than 0.6 [32]. Additional description of the survey instrument development (including cognitive testing and pretesting) and telephone interview procedures have been described previously [32].

### Main outcomes

#### Body Mass Index (BMI)

Participants self-reported height and weight; body mass index (BMI) was calculated using weight/height^2^ (kg/m^2^) and was dichotomized as under/normal/overweight (BMI <30 kg/m^2^) and obese (BMI > = 30kg/m^2^) [37].

#### Dietary intake

Measurements of fruits and vegetables were based on the 2011 Behavioral Risk Factor Surveillance Survey [38]. Measurements of sugar consumption were based on the California Health Interview Survey [39], which asks about foods such as cookies, cakes, pies, brownies, ice cream, and frozen desserts and beverages such as non-diet soda/pop, sweetened tea and coffee beverages, and sweetened fruit drinks. These items asked about food consumption in the previous month. Response options were open-ended, allowing the participant to report the number of times per day, week, or month they consumed the given food item, creating continuous variables. Finally, frequency of fast food consumption in the previous week was measured using one item, which asked: “In the past 7 days, how many times did you eat fast food? Include fast food meals eaten at work, at home, or at fast-food restaurants, carryout or drive through” [39].

#### Physical activity

Selected items from the International Physical Activity Questionnaire (IPAQ) were used to collect self-reported PA [40, 41]. These included occupational, transportation and leisure-time physical activities. IPAQ has been extensively tested internationally for reliability (Spearman’s rho clustered ~0.8) and validated with objective PA measures (median rho ~0.3). This measure has found to be comparable to most other self-reported measures [41]. Based on the participant’s total PA, measured with the IPAQ, the sample was dichotomized based on the Physical Activity Guidelines for Americans (≥150 minutes of PA per week) [42].

### Main exposure

#### Workplace Social/Organizational Environment

One item, from the California Check for Health measure [43] assessed whether the participant felt their company valued their health. Items assessing co-worker behaviors and modeling were developed for this survey and asked the participants the extent to which their co-workers served as role models for healthy food and activity behaviors; other items assessed the frequency to which participants observed co-workers engaging in healthy behaviors such as eating fruits and vegetables or being physically active.

#### Covariates and socio-demographic variables

Since previous research has shown that demographic characteristics and workplace size might be related the behaviors [23–29] and/or the workplace characteristics [30, 31] under investigation, we explored several characteristics. Participants’ self-reported demographic characteristics are available in Table 1. Race categories were condensed to White, Black or African American, and all others. Reported worksite size was divided into tertiles (0–49, 50–199, and 200 or more) based on the distribution of the sample. Annual household income was reported in categories <40,000 U.S. dollars to more than 75,000 U.S. dollars at 10,000 increments, but was dichotomized based on our previous work in this population [44]. Participants reported age, which was divided into tertiles (21–44; 45–54; 55–65) based on the structure of the sample, and sex (male/female).

**Table 1:** Social/Organizational, Diet, physical activity, and Obesity variables and demographics %^†^ (n), Missouri, USA n = 1,338.

	Race			Worksite Size			Income		Age			Sex		Total
	White	Black/AA	Other	0–49	50–199	200+	0-$29K	$30K+	21–44	45–54	55–65	Female	Male	
Total	63.6% (1127)	29.1% (516)	6.2% (110)	30.6% (542)	30.2% (536)	34.6% (613)	18.3% (325)	75.6% (1340)	33.9% (601)	32.7% (580)	32.1% (568)	68.6% (1215)	31.4% (556)	
Company values health														
Agree	87.3% (959)	81.4% (407)	87.4% (90)	84.2% (441)	84.0% (436)	87.8% (525)	78.7% (248)	87.1% (1134)	82.6% (483)	85.3% (482)	88.8% (485)	84.5% (993)	87.7% (476)	83.0% (1470)
Total	1098	500	103	524	519	598	315	1302	585	565	546	1175	543	1719
Chi-sq p	0.006*			0.122			<.001*		0.011*			0.085		
Role models for food														
Agree	55.7% (604)	55.9% (279)	59.8% (64)	55.3% (291)	57.3% (295)	55.8% (332)	51.1% (161)	56.3% (728)	49.90% (291)	59.40% (335)	57.90% (313)	57.4% (679)	52.1% (274)	53.8% (954)
Total	1085	499	107	526	515	595	315	1293	583	564	541	1182	526	1709
Chi-sq p	0.711			0.801			0.097		0.002*			0.04*		
Role models for physical activity														
Agree	58.5% (635)	55.0% (275)	59.0% (62)	55.6% (297)	57.8% (293)	58.8% (350)	54.9% (173)	57.7% (747)	53.7% (312)	62.0% (348)	56.0% (305)	58.1% (683)	55.9% (298)	55.4% (982)
Total	1086	500	105	534	507	595	315	1294	581	561	545	1175	533	1709
Chi-sq p	0.406			0.544			0.367		0.014*			0.39		
See co-workers eating fruits and vegetables														
Agree	82.9% (927)	81.6% (420)	77.1% (84)	80.9% (435)	80.3% (428)	85.2% (520)	73.5% (236)	84.3% (1125)	79.8% (477)	83.5% (480)	83.0% (468)	83.9% (1015)	78.1% (429)	81.5% (1445)
Total	1118	515	109	538	533	610	321	1334	598	575	564	1210	549	1760
Chi-sq p	0.287			0.053			<.001*		0.198			0.004*		
See co-workers doing physical activity														
Agree	32.4% (362)	55.9% (287)	43.1% (47)	30.3% (163)	42.1% (224)	47.5% (289)	53.2% (173)	36.5% (484)	43.3% (258)	40.7% (234)	35.0% (197)	40.7% (491)	38.2% (210)	39.6% (702)
Total	1116	513	109	538	532	608	325	1327	596	575	563	1205	550	1756
Chi-sq p	<.001*			<.001*			<.001*		0.013*			0.309		
See co-workers doing physical activity during work breaks														
Agree	40.9% (458)	57.1% (292)	47.2% (51)	35.3% (190)	47.5% (252)	54.8% (333)	48.8% (158)	45.3% (601)	42.2% (250)	51.4% (296)	44.3% (250)	46.6% (560)	45.1% (249)	45.7% (810)
Total	1119	511	108	538	530	608	324	1328	593	576	564	1203	552	1756
Chi-sq p	<.001*			<.001*			0.256		0.004*			0.574		
Fruit daily														
> = 1/d	66.5% (750)	56.4% (291)	62.7% (69)	62.7% (340)	62.3% (334)	64.9% (398)	52.9% (172)	65.6% (879)	56.9% (342)	64.8% (376)	68.3% (388)	64.6% (785)	60.6% (337)	63.3% (1122)
Total	1127	516	110	542	536	613	325	1340	601	580	568	1215	556	1772
Chi-sq p	<.001*			0.609			<.001*		<.001*			0.105		
Vegetables daily														
> = 1/d	81.2% (915)	65.1% (336)	78.2% (86)	76.2% (413)	76.1% (408)	76.7% (470)	64.9% (211)	78.8% (1056)	71.9% (432)	76.2% (442)	81.3% (462)	77.6% (943)	73.9% (411)	76.5% (1355)
Total	1127	516	110	542	536	613	325	1340	601	580	568	1215	556	1772
Chi-sq p	<.001*			0.972			<.001*		0.001*			0.089		
Sugars daily														
> = 1 /d	50.2% (566)	65.9% (340)	66.4% (73)	61.3% (332)	53.4% (286)	54.5% (334)	65.8% (214)	53.4% (715)	63.6% (382)	55.0% (319)	48.6% (276)	53.9% (655)	60.3% (335)	55.9% (991)
Total	1127	516	110	542	536	613	325	1340	601	580	568	1215	556	1772
Chi-sq p	<.001*			0.017*			<.001*		<.001*			0.013*		
Times eat fast food														
2+x/wk	41.4% (467)	58.4% (301)	35.5% (39)	49.9% (270)	43.7% (234)	46.8% (287)	51.9% (168)	44.4% (595)	51.8% (311)	46.7% (271)	38.9% (221)	45.5% (552)	46.8% (260)	45.8% (812)
Total	1127	515	110	541	536	613	324	1340	600	580	568	1214	556	1771
Chi-sq p	<.001*			0.121			0.016*		<.001*			0.612		
Physical Activity Level														
150+ min	80.2% (869)	76.3% (376)	81.0% (85)	80.3% (415)	80.9% (412)	75.5% (451)	82.4% (252)	78.4% (1016)	80.6% (470)	79.7% (445)	77.3% (416)	76.1% (886)	85.6% (458)	75.9% (1345)
Total	1083	493	105	517	509	597	306	1296	583	558	538	1164	535	1700
Chi-sq p	0.177			0.054*			0.125		0.375			<.001*		
Obesity														
BMI≥30	29.7% (324)	46.5% (227)	30.3% (30)	31.9% (169)	34.8% (179)	37.0% (214)	41.5% (129)	33.1% (429)	34.5% (202)	35.4% (196)	33.9% (185)	35.3% (406)	33.0% (180)	33.1% (586)
Total	1092	488	99	529	515	579	311	1295	585	553	545	1150	546	1696
Chi-sq p	<.001*			0.215			0.005*		0.871			0.344		

^†^% within demographic characteristic

^‡^In the last 12 months, how often were you concerned about having enough money to eat nutritious meals? Would you say…

## Analysis

The social/organizational environment variables were dichotomized (strongly agree and agree vs disagree/strongly disagree). Diet behaviors included eating fruits (at least 1 time per day), eating vegetables (at least 1 time per day), eating snack foods, and eating fast food (at least two times per week). These variables were dichotomized based on the low prevalence of fruit and vegetable intake among U.S. adults [45, 46], the contribution of fast-food to U.S. diets [47], as well as the distribution in the data. PA behaviors included reporting at least 150 minutes of PA per week [42] and obesity (as assessed by BMI) was dichotomized at 30 (with BMI≥30 being the definition of obesity) [37]. Bivariate associations were explored between the social/organizational environment, diet and PA behaviors, obesity, and demographics. Logistic regression models explored the association between social/organizational environment variables and diet, PA, and obesity; these were conducted with and without adjustment for demographic factors (race, employer size, age, sex, and income). Variables for adjustment were selected based on the associations in the bivariate analyses and those common in the nutrition epidemiology literature. To be consistent, we included the same adjustment variables in all analyses.

## Results

### Bivariate analyses

Table 1 summarizes the population demographics as well as participation in health behaviors by race, employer size, age, sex, and income. In bivariate analyses, White, older, and higher income participants reported healthier diets, and males tended to be more active (Table 1). Lower income individuals were more likely to be obese compared to higher income individuals; obesity was highest among those reporting their race as Black or African-American, than those of other races, followed by Whites.

There were differences in social/organizational variables by demographic characteristics. Agreement with the statement the ‘company values my health’ was highest among Whites/those of other races, older employees, and higher income workers, compared to Black or African-Americans, younger workers, and lower income employees, respectively. Respondents at larger employers, those earning higher income, and females were more likely to report seeing co-workers eating fruits and vegetables. However, those reporting seeing co-workers doing PA were more likely to be lower income. Non-White workers were more likely to report seeing co-workers doing PA and engaging in PA during breaks at work. Those reporting seeing role models for positive food behaviors tended to be older workers and female. As worksite size increased, the frequency of reporting seeing co-workers doing several types of healthy behaviors (eat fruits and vegetables, doing PA, and doing PA on breaks at work) increased.

### Multivariate analyses

In multivariate analyses, employees who reported seeing co-workers eating fruits and vegetables were more likely to report eating at least one vegetable per day (aOR = 1.43, 95% CI: 1.06–1.91) and at least one fruit per day, however, this was not significant after adjustment (aOR = 1.31, 95% CI: 0.99–1.71) (Table 2). Similarly, workers who reported they see co-workers engaging in PA were more likely to report higher levels of PA (aOR 1.56, 95% CI: 1.19–2.05).

**Table 2:** Crude and adjusted* logistic regression of diet, physical activity, and obesity variables and social/organizational variables OR (95% CI), Missouri, USA n = 1,338.

	≥1 Fruit/day	≥1 /Vegetables/day	<1 Sugars/d	≥2 Fast food/week	Physical Activity Level 150+ min	BMI≥30
Company values health						
Crude	1.32(1.01–1.74)	1.37(1.01–1.85)	1.11(0.85–1.46)	0.76(0.58–1.00)	1.56(1.14–2.13)	0.68(0.51–0.90)
Adj*	1.25(0.93–1.67)	1.33(0.97–1.84)	0.98(0.73–1.32)	0.84(0.63–1.12)	1.54(1.09–2.16)	0.73(0.54–0.98)
Role models for food						
Crude	1.25(1.03–1.53)	1.02(0.81–1.28)	0.90(0.75–1.10)	1.13(0.93–1.37)	-	1.18(0.96–1.45)
Adj*	1.13(0.92–1.40)	0.95(0.75–1.21)	0.83(0.67–1.02)	1.15(0.94–1.42)	-	1.13(0.91–1.41)
See co-worker eating fruits and vegetables						
Crude	1.43(1.12–1.83)	1.60(0.55–2.10)	1.05(0.82–1.35)	1.14(0.89–1.46)	-	0.92(0.71–1.19)
Adj*	1.31(0.999–1.71)	1.43(1.06–1.91)	0.90(0.69–1.18)	1.22(0.93–1.59)	-	0.94(0.71–1.24)
Crude	-	-	-	-	1.20 (0.94–1.52)	0.99(0.81–1.22)
Adj*	-	-	-	-	1.20(0.93–1.54)	0.99(0.80–1.23)
See co-worker doing physical activity						
Crude	-	-	-	-	1.39 (1.08–1.78)	1.11 (0.90–1.36)
Adj*	-	-	-	-	1.56 (1.19–2.05)	1.02(0.81–1.28)
See co-worker physical activity during work break						
Crude	-	-	-	-	1.01(0.79–1.27)	1.36(1.11–1.66)
Adj*	-	-	-	-	0.98(0.76–1.27)	1.23(0.99–1.53)

*Adjusted (Race, Employer size, Age, Sex, and Income)

The presence of role models for healthy food choices was associated with the likelihood of eating at least one fruit per day; however, this was not significant after adjustment (aOR = 1.13, 95% CI: 0.92–1.40). Similarly, workers who reported they agree with the statement that their company values their health were more likely to report eating at least one fruit and at least one vegetable per day. However, this was only significant before adjustment (Fruit: aOR = 1.25, 95% CI: 0.93–1.67; vegetables: aOR = 1.33, 95% CI: 0.97–1.84). Workers agreeing with this statement were also less likely to be obese before and after adjustment (aOR = 0.73, 95% CI: 0.54–0.98), and more likely to report higher levels of PA before and after adjustment (aOR = 1.54, 95% CI: 1.09–2.16).

## Discussion

There were important differences between reports of social/organizational characteristics by those with different demographic characteristics. Our exploratory study suggests that reports of seeing co-workers doing several types of healthy behaviors (eating fruits and vegetables, doing PA, and doing PA on breaks at work) seemed to be related to a number of outcomes including fruit and vegetable intake and PA, and these all increased as worksite size increased. It is possible that larger work places offer more resources and greater numbers and diversity of co-workers, thus allowing employees more opportunities to observe their co-workers engaging in healthy behaviors. This fits with behavior theories indicating the importance of norms [14–16]. There were additional differences by gender, race, and income in the reporting of these characteristics. Further, we found associations between agreement with the statement that the company values the employee’s health and important behavior/obesity outcomes, indicating employees agreeing with the statement report obesity-preventive behaviors, though these were somewhat reduced with adjustment. It is possible that these companies provide more resources toward employee health and well-being. As with seeing co-workers engaging in healthy behaviors, report of agreement with this statement that the company valued the participant’s health also varied by demographic characteristics, for example, reports of agreement were less frequent among Blacks, those reporting lower income, and younger workers. This suggests a culture of healthy behaviors in a workplace is related to worker health behaviors [48].

Others have found positive relationships between normative eating and activity behaviors (i.e., perceived behaviors of co-workers) and dietary and PA behaviors [11, 22, 49]. Further, among hospital employees, Lemon et al. [11] found relationships between organizational commitment to employee health and BMI and Sliter et al. [49] found that the workplace climate for healthy weight maintenance was uniquely predictive of most individual variables, diet quality and PA, in particular. However, these results have not been consistent in the literature. Basen-Engquist et al. [50] did not find relationships between organizational climate and employee behaviors. Our results indicated stronger relationships between normative health behaviors and dietary behaviors than those for PA. Lemon et al. found similar results and suggested this may be a result of the routine activity of eating, but not exercising, as a part of the workday [11].

The current study found important differences in perceptions of workplace support based on demographic characteristics; for example women were more likely to report seeing co-workers eating fruits and vegetables than men, and White, older, and higher income workers were more likely to feel the company values health than younger, Black/African American or other, or lower income employees. The discrepancy by gender has been seen in previous studies [11, 22]. In a study of hospital employees, women had higher perceptions of coworker healthy eating behaviors than men, and Hispanics and Asians/others had higher perceptions than non-Hispanic Whites. Further, non-Hispanic Blacks had higher perceived organizational commitment to employee health than non-Hispanic whites, which is the opposite of what we found in the current sample. Other studies have not found differences based on ethnicity [22].

This study has several implications. First, it suggests there is a need for further study, including development and evaluation of intervention strategies promoting changes in social norms in the workplace around eating and PA. This work also suggests that employees may benefit from greater visibility for positive health behaviors, perhaps through management support. For example, managers might highlight a particular employee or group of employees’ participation in a positive fashion or encourage participation. Similarly, there may be benefit to rewarding/encouraging health behaviors so they become more pervasive as well as promotion of early adopters and champions. Finally, this work points to the potential for implementing and evaluating policy change at worksites aimed at creating a food and PA environment that fosters modeling. Such changes might include informal policies specifying healthy foods be served at workplace-sponsored events, such employees would observe each other eating healthy foods or that meetings should include exercise breaks, where employees might observe their peers being active. Future studies, using longitudinal and experimental designs should investigate these strategies.

This study has limitations worth noting. From this cross-sectional study, it is not possible to determine the direction of causality; for example if perceived organizational support for employee health causes lower BMIs among employees or if employees with lower BMIs might be drawn to such workplaces or may be those who are more aware of the supports an organization offers. Further, the relationship between norms and behaviors may suggest people notice those more like themselves, rather than suggesting that the diet and PA behavior of some co-workers influence those of others. Additionally, the workplace perceptions and measures for BMI, diet, and PA were collected by self-report, which are subject to bias as well as inaccuracy of reporting. Another limitation is the use of the IPAQ to assess physical activity. Specifically, it is not possible to assess the intensity of walking participants engaged in. For this analysis we included all walking as contributing to PA. Thus, we may have overestimated the PA level of the population; IPAQ overestimates PA when compared with objective measures [51, 52]. It is also worth noting that IPAQ was primarily developed for cross-national surveillance not estimating guideline adherence or intervention effects [41, 53, 54]. There is the potential for bias based on who responded to the survey, particularly given the low response rate and the use of only landline phone numbers. Further, the survey was conducted among working populations in Missouri, and therefore may not generalize to the non-working population.

### Conclusions

This research suggests that social/organizational characteristics of the workplace environment may be related to nutrition and PA behaviors and obesity. Social/organizational characteristics were particularly related to feeling the company values the workers’ health and to seeing co-workers engaging in healthy behaviors. These findings point to the potential for intervention targets such as environment and policy changes.

## Supporting Information

S1 Appendix Survey item wording. (DOCX)
