# Supplementary material for: Workplace Social and Organizational Environments and Healthy-Weight Behaviors
Source: PLoS One. 2015 Apr 28;10(4):e0125424. doi: 10.1371/journal.pone.0125424 (PMC4412705; doi:10.1371/journal.pone.0125424)
Supplement: S1 Appendix — (DOCX) [file pone.0125424.s001.docx]

Supporting Information Appendix S1. Survey item wording.

| Item wording | Response options | Reference |
| --- | --- | --- |
| **ORGANIZATIONAL CHARACTERISTICS and SOCIAL ENVIRONMENT SUPPORTS** | | |
| About how many employees work for your company at your location?  *[Interviewer note: This means everyone who works for the company at that worksite campus, not just those in the same department or floor or division.]* | [open ended]  If ‘don’t know’, prompt to code categories (<100, <50, <25, <200, <500, <1000) | NEW^a^ |
| My company (or organization) values healthy workers. | strongly disagree, disagree, agree, or strongly agree | NEW^a^ |
| My co-workers are good role models for making healthy food choices. | strongly disagree, disagree, agree, or strongly agree | NEW^a^ |
| My co-workers are good role models for a physically active lifestyle. | strongly disagree, disagree, agree, or strongly agree | NEW^a^ |
| *Please indicate how often you see your co-workers doing each of the following:* | | |
| I see co-workers eating fruits and vegetables. Would you say: | Never, Rarely, Sometimes, Often | NEW^a^ |
| I see coworkers walking, biking, or taking public transportation to get to work. Would you say: | Never, Rarely, Sometimes, Often | NEW^a^ |
| I see coworkers being physically active during their work breaks. Would you say: | Never, Rarely, Sometimes, Often | NEW^a^ |
| **PHYSICAL ACTIVITY** | | |
| **JOB-RELATED**  *The first section is about your work. Do not include unpaid work you might do around your home, like housework, yard work, general maintenance, and caring for your family. The next questions are about all the physical activity you did in the* ***last 7 days*** *as part of your paid work. This does not include traveling to and from work* | | |
| During the **last 7 days**, on how many days did you do **vigorous** physical activities like heavy lifting, digging, heavy construction, or climbing up stairs **as part of your work**? Think about only those physical activities that you did for at least 10 minutes at a time. | Yes, no | IPAQ^b^[1] |
| How much time did you usually spend on one of those days doing **vigorous** physical activities as part of your work? | _____hours/day, _____minutes/day | IPAQ ^b^[1] |
| Again, think about only those physical activities that you did for at least 10 minutes at a time. During the **last 7 days**, on how many days did you do **moderate** physical activities like carrying light loads **as part of your work**? Please do not include walking. | Yes, no | IPAQ ^b^[1] |
| How much time did you usually spend on one of those days doing **moderate** physical activities as part of your work? | _____hours/day, _____minutes/day | IPAQ ^b^[1] |
| During the **last 7 days**, on how many days did you **walk** for at least 10 minutes at a time **as part of your work**? Please do not count any walking you did to travel to or from work. | Yes, no | IPAQ ^b^[1] |
| How much time did you usually spend on one of those days doing **walking** as part of your work? | _____hours/day, _____minutes/day | IPAQ ^b^[1] |
| **TRANSPORTATION**  *These questions are about how you traveled from place to place, including to places like work, stores, movies, and so on.*  *Now think only about the* ***bicycling*** *and* ***walking*** *you might have done to travel to and from work, to do errands, or to go from place to place.* | | |
| During the last 7 days, on how many days did you bicycle for at least 10 minutes at a time to go from place to place? | Yes, no | IPAQ ^b^[1] |
| How much time did you usually spend on one of those days to bicycle from place to place? | _____hours/day, _____minutes/day | IPAQ ^b^[1] |
| During the last 7 days, on how many days did you walk for at least 10 minutes at a time to go from place to place? | Yes, no | IPAQ ^b^[1] |
| How much time did you usually spend on one of those days walking from place to place? | _____hours/day, _____minutes/day | IPAQ ^b^[1] |
| **RECREATION, SPORT, AND LEISURE-TIME**  *This section is about all the physical activities that you did in the* ***last 7 days*** *solely for recreation, sport, exercise or leisure. Please do not include any activities you have already mentioned.* | | |
| Not counting any walking you have already mentioned, during the last 7 days, on how many days did you walk for at least 10 minutes at a time in your leisure time? | Yes, no | IPAQ ^b^[1] |
| How much time did you usually spend on one of those days walking in your leisure time? | _____hours/day, _____minutes/day | IPAQ ^b^[1] |
| *Think about only those physical activities that you did for at least 10 minutes at a time.* | | |
| During the last 7 days, on how many days did you do vigorous physical activities like aerobics, running, fast bicycling, or fast swimming in your leisure time? | Yes, no | IPAQ ^b^[1] |
| How much time did you usually spend on one of those days doing vigorous activity in your leisure time? | _____hours/day, _____minutes/day | IPAQ ^b^[1] |
| During the last 7 days, on how many days did you do moderate physical activities like bicycling at a regular pace, swimming at a regular pace, and doubles tennis in your leisure time? | Yes, no | IPAQ ^b^[1] |
| How much time did you usually spend on one of those days doing moderate activity in your leisure time? | _____hours/day, _____minutes/day | IPAQ ^b^[1] |
| **EATING BEHAVIOR** | | |
| **FRUIT & VEGETABLE INTAKE**  *These next questions are about the fruits and vegetables* ***you*** *ate or drank during the past 30 days. Please think about all forms of fruits and vegetables including cooked or raw, fresh, frozen or canned. Please think about all meals, snacks, and food consumed at home and away from home.* | | |
| During the past month, how many times per day, week or month did you drink 100% PURE fruit juices? Do not include fruit-flavored drinks with added sugar or fruit juice you made at home and added sugar to. Only include 100% juice. | [open ended]  per day, per week, per month | BRFSS 2011^c^[2] |
| During the past month, not counting juice, how many times per day, week, or month did you eat fruit? Count fresh, frozen, or canned fruit | [open ended]  per day, per week, per month | BRFSS 2011 ^c^[2] |
| During the past month, how many times per day, week, or month did you eat cooked or canned beans, such as refried, baked, black, garbanzo beans, beans in soup, soybeans, edamame, tofu or lentils. Do NOT include long green beans. | [open ended]  per day, per week, per month | BRFSS 2011 ^c^[2] |
| During the past month, how many times per day, week, or month did you eat dark green vegetables for example broccoli or dark leafy greens including romaine, chard, collard greens or spinach? | [open ended]  per day, per week, per month | BRFSS 2011 ^c^[2] |
| During the past month, how many times per day, week, or month did you eat orange- colored vegetables such as sweet potatoes, pumpkin, winter squash, or carrots? | [open ended]  per day, per week, per month | BRFSS 2011 ^c^[2] |
| Not counting what you just told me about, during the past month, about how many times per day, week, or month did you eat OTHER vegetables? Examples of other vegetables include tomatoes, tomato juice or V-8 juice, corn, eggplant, peas, lettuce, cabbage, and white potatoes that are not fried such as baked or mashed potatoes. | [open ended]  per day, per week, per month | BRFSS 2011 ^c^[2] |
| **ADDED SUGARS**  *Now think about the foods you ate or drank during the past month, that is, the past 30 days, including meals and snacks.* | | |
| [During the past month,] how often did you drink regular soda or pop that contains sugar? Do not include diet soda. | [open ended]  per day, per week, per month | CHIS 2009^d^[3] |
| [During the past month,] how often did you drink coffee or tea with sugar or honey added? Do not include drinks with things like Splenda or Equal. Include pre-sweetened tea and coffee drinks such as Arizona Iced Tea and Frappuccino. | [open ended]  per day, per week, per month | CHIS 2009 ^d^[3] |
| [During the past month,] how often did you drink sports or energy drinks such as Gatorade, Red Bull, and Vitamin water? Do not include diet or sugar-free kinds. | [open ended]  per day, per week, per month | CHIS 2009 ^d^[3] |
| [During the past month,] how often did you drink sweetened fruit drinks such as Kool-aid, cranberry drink, and lemonade? Include fruit drinks you made at home and added sugar to. | [open ended]  per day, per week, per month | CHIS 2009 ^d^[3] |
| [During the past month,] how often did you eat cookies, cake, pie, or brownies? Do not include sugar-free kinds. | [open ended]  per day, per week, per month | CHIS 2009 ^d^[3] |
| [During the past month,] how often did you eat ice cream or other frozen desserts? Do not include sugar-free kinds. | [open ended]  per day, per week, per month | CHIS 2009 ^d^[3] |
| **FAST FOOD** | | |
| Now think about the past week. In the past 7 days, how many times did you eat fast food? Include fast food meals eaten at work, at home, or at fast-food restaurants, carryout or drive through. | [open ended] | CHIS 2009 ^d^[3] |
| **DEMOGRAPHICS and HEALTH** | | |
| How old are you? | [open ended] |  |
| Are you male or female? | MALE, FEMALE, REFUSE, DON’T KNOW |  |
| Do you consider yourself Hispanic/Latino? | Yes, no |  |
| Which one or more of the following would you say is your race? | White, Black or African American, Asian, Native Hawaiian or Other Pacific Islander, American Indian or Alaska Native, Other [specify] |  |
| What is your height? | FEET and INCHES, or METERS and CENTIMETERS |  |
| What is your current weight? | Pounds or kilograms |  |
| Is your annual household income from all sources: | < $40,000, <$30,000, <$20,000, <$10,000, <$50,000, <$60,000, <$70,000, >$75,000 |  |

^a^NEW=newly developed for this survey

^b^IPAQ=International Physical Activity Questionnaire

^c^BRFSS 2011=2011 Behavioral Risk Factor Surveillance Survey

^d^CHIS 2009=California Health Interview Survey

1 The International Physical Activity Questionnaire (Short Last 7 Days Telephone Format), Revised 2002. Or is it https://sites.google.com/site/theipaq/.

2 Centers for Disease Control and Prevention (CDC). Behavioral Risk Factor Surveillance System Survey Questionnaire. Atlanta, Georgia: U.S. Department of Health and Human Services, Centers for Disease Control and Prevention, 2011.

3 CHIS California Health Interview Survey, 2009. *Los Angeles (CA): UCLA Center for Health Policy Research*.
